# The Early Diagnosis of Spinal Caries, with Remarks on Treatment

**Published:** 1887-11

**Authors:** Hunter P. Cooper

**Affiliations:** Professor of General and Medical Chemistry in the Atlanta Medical College; Atlanta, Ga.


THE ATLANTA MEDICAL AND SURGICAL JOURNAL.

VOL. IV. NOVEMBER, 1887. No. 9.

### Original Communications.

### THE EARLY DIAGNOSIS OF SPINAL CARIES WITH REMARKS ON TREATMENT.*

BY HUNTER P. COOPER, M. D., PROFESSOR OF GENERAL AND MEDICAL CHEMISTRY IN THE ATLANTA MEDICAL COLLEGE.

Every pitiable, shrunken hunch-back that we pass on the street is a *raison d'être* for this paper. How well do we know the sight, for unfortunately it is very common. The huge head, sunken between misshapen shoulders ; the pallid face, with deep lines of suffering drawn across it by months and years of ceaseless pain and anguish ; the unsightly hump on the back, diminishing, almost by half, man's normal stature and totally destroying ease and grace of carriage.

The mental suffering of these poor unfortunates is perhaps even greater than their bodily pain. Throughout life the eyes of the curious and vulgar make them a target ; every man who passes them silently and unconsciously taunts them by his erect and manly carriage. Their lot is all the harder because of its utter hopelessness.

* Read before the Atlanta Society of Medicine.

I make no apology for writing on an old subject and saying things that have been said before, for on subjects like this every generation should not only avail itself of the wisdom of its predecessors, but should do its own reading, writing and thinking. There is, indeed, a voluminous literature on Pott's disease of the spine, the most valuable part of which comes from our own countrymen, but it is also true that this literature has been much neglected, and some may read what I have to say who would not read more elaborate treatises.

It is a disease which few physicians have carefully studied, and when a case of it falls into the hands of one who has had little or no experience with it, he often fails to recognize it until it is too late, and the spine is hopelessly distorted.

The general practitioner rarely meets with the disease, and the descriptions of it in the text-books are often misleading, and hence the early symptoms are apt to escape attention. They pass for "growing pains" in children and for "rheumatism" in adults; a liniment is prescribed and the patient is dismissed. The symptoms quietly continue, gradually growing worse until the friends call the doctor's attention to a deformity of the spine. Then of course the diagnosis is perfectly simple, and proper treatment instituted; but valuable time has been lost, and the deformity increases month by month despite mechanical restraint. I desire to make the early diagnosis of Pott's disease so clear that any physician can detect it without waiting for a large prominence to form on the spine. Gibney, of New York, and Bradford, of Boston, have done as much in simplifying the diagnosis, as Sayre has done in improving the therapeutics of spinal caries.

My practical knowledge of this disease was gained during a year's service as interne in the Hospital for the Ruptured and Crippled in New York. During this period I saw a large number of patients in the various stages of spinal disease, and I derived much valuable instruction from my friend, Dr. Gibney, who was then serving his fourteenth year as house surgeon.

I shall quote in the first place a case reported by Dr. Gibney in an article in the *Boston Medical and Surgical Journal* in 1882.

I use this case because it is very similar to one narrated below in my own practice, and it points an excellent moral :

Fred. C., aged 32, applied June 22, 1881, near the end of the morning clinic, for relief of distressing pains, which he referred to the thoracic walls and hypogastrium. I made a hurried examination, finding a very tender dorsal spine, and tenderness over the intercostal nerves. Fowler's solution and counter-irritation were ordered, and he was given explicit instructions to call in a few days for a more thorough examination. His first visit after the above date was on December 24, 1881, when he came walking into the office stooping over like an old man, and bearing the following note from my friend, Dr. Ripley:

"DEAR DOCTOR—You saw this man four months ago, *he says.* I should like your more mature opinion. Truly yours, J. H. R."

It did not require any mature opinion now for a diagnosis; the kyphosis spoke for itself. . . . . . . His spinal tenderness on the first visit misled me, and this fact, taken in connection with the lack of time, prevented me from making an examination, which would, without doubt, have led to a correct diagnosis.

I do not mean to cast any reflection on Dr. Gibney, for I am sure he would have made a diagnosis had time allowed a careful examination, or had the patient returned as instructed. The case illustrates the importance of making careful examinations of patients presenting spinal symptoms. The following case from my own practice is strikingly like the above:

Mr. D., aged 35, merchant, came to me from a neighboring town in December, 1886, with this history: Ever since April, 1886, he has suffered from very distressing pains in the back and in the left side, extending around to the abdomen. In August his suffering became much more intense, and has continued to grow worse. He cannot rest at night on account of pain. For the past two or three months he has been walking with a stiff, awkward gait, the right shoulder being elevated higher than the left. During all his sickness his digestion has been very bad. On examination the patient is found to be well nourished, but his flesh is soft and flabby and complexion very sallow. When he stands, with his heels together, the right shoulder is seen to be considerably higher than the left and slightly advanced. In stooping he does not bend the spine at all. When he walks it is with the characteristic gait of Pott's disease—that is, the head and body are held perfectly rigid, and the patient advances with a careful step and watchful eye, lest he should accidentally jolt his spine. Examination of the spine reveals a lateral curvature (slight) in the dorsal region, with the convexity to the right; with this there is a slight rotation of the bodies of the vertebræ, throwing the right shoulder and right side of the chest forward. There is undue prominence of the tenth dorsal spinous process; this coincides with the seat of greatest pain. There is marked tenderness over the lower dorsal and all the lumbar spinous processes, this being most acute over the tenth dorsal. Tenderness is also present over the umbilical and left hypochondriac regions. Diagnosis, Pott's disease; treatment, spinal jacket and internal medication.

A plaster of Paris jacket was applied December 14, 1886, Drs. Gray and Lind kindly assisting me. The patient improved considerably while wearing this jacket, but it was not a good fit, and another was applied January 29, '87, Drs. Harris and Woodward being present and lending their aid. Since that date (to make a long story short) the patient has been like a different creature, has gained flesh and strength, has always a good appetite and digestion, has been *almost entirely* free from pain, and has attended constantly to his business. He is now wearing a jacket applied August 6th, two months ago. At that time the lateral curvature had entirely disappeared, the two shoulders were practically on the same level, and the dorsal kyphosis was considerably diminished. Dr. N. O. Harris has assisted me in applying three jackets, and can testify to the patient's great improvement.

This patient suffered for months from pain in the back, in the side and in the abdomen before any change in his gait appeared. During this period he was treated for rheumatism, of course without success. When he came to me the diagnosis was perfectly clear, because, in addition to well-marked symptoms, he presented a dorsal kyphosis. I am satisfied, however, that a diagnosis could have been reached months before had a careful examination been made and sufficient weight attached to the symptoms he then had.

DIAGNOSIS IN EARLY STAGE.

As an evidence of how rarely the diagnosis is made early in the disease, Gibney reports that out of 196 cases of spinal caries that came under his observation, only 14 were free from spinal deformity when first seen by him; and Sayre, in his work on orthopedic surgery, says that only three out of 225 cases were without deformity when they first came under his treatment. Now as the vast majority of such cases are under the charge of the family physician for a considerable time before drifting into the hands of the specialist, these figures show how rarely the general practitioner reaches a diagnosis previous to the stage of deformity.

The symptoms to be relied on for an early diagnosis are these: Pain, gait, rigidity of the spine, and the general condition, appearance and behavior of the patient. An abscess will also be found sometimes with these early symptoms. The family and personal history of the patient must also be carefully inquired into. A traumatism can nearly always be discovered in the patient's personal history, but this will have much or little weight in influencing the diagnosis, according to the physician's views on the etiology of Pott's disease. If he is a follower of Sayre, he will find in a very slight traumatism a sufficient cause for spinal caries. If, however, he believes with Gibney and Treves, that a scrofulous or tubercular tendency is the most important etiological factor, he will not attribute much weight to a trifling traumatism unless, at the same time, the patient presents some evidence of scrofula or tuberculosis, hereditary or acquired.

The disease in its incipiency presents some differences in adults and in children.

IN ADULTS the symptoms are often obscure, resembling rheumatism, neuralgia or lumbago. A persistent intercostal pain, especially if accompanied by pain or aching in the back and abdominal pains should always lead us to make a careful examination of the spine, for these symptoms may continue a long time before any deformity. On stripping the patient and making pressure successively along the different vertebræ, we often find one or more spinous processes very tender. I know this statement is at variance with the authorities, but it has nevertheless been the rule in the cases of Pott's disease I have seen *in adults.* In children this tenderness is rarely present. In the case quoted above from Dr. Gibney's practice, he says, "His spinal tenderness on the first visit misled me." This patient was an adult and presented spinal tenderness, a symptom so little regarded now as a symptom of spinal caries that Dr. Gibney was misled by its presence. Why this spinal tenderness should be found in adults and not in children suffering with Pott's disease is more than I can explain; yet I believe it is true that in the incipient stage of spinal disease in *grown people* this symptom of spinal tenderness is very generally present. In addition to this, there is in adults another peculiarity, viz., as to the history of traumatism. In adults there is much oftener than in children a history of *violent* traumatism. These constitute the differences between the early symptoms in adults and in children.

IN CHILDREN there is usually a history for a longer or shorter time of irregular pains, occurring according to the location of the carious process in the arms or shoulders, chest or abdomen. The child does not sleep well, suffering at night as well as in the day. During the day the little patient does not play as in health, frequently stopping to take a rest. The prone position is often assumed in order to ease the spine. As the child walks about the room, it is apt to make use of the chairs and other furniture for support, leaning its weight first on one and then on the other. If the disease is in the cervical or high up in the dorsal region, the child frequently lays its little head down in its mother's lap, or upon the bed, to relieve the incessant pressure upon the diseased bone.

The gait of the patient is one of the most characteristic symptoms; the peculiarity of the gait is due to two factors: first, the rigidity of the spine; second, the desire of the patient to prevent any jolting of the spine. The patient advances with a careful and even apprehensive expression; the body is held perfectly rigid, and the feet are placed on the floor softly and cautiously, so as to transmit to the spine as little abrupt motion as possible. If the disease is in the cervical or upper dorsal region, there is frequently either a slight torticollis, or opisthotonos, due to muscular contraction; this gives an additional peculiarity to the patient's appearance as he walks.

In some cases the patient advances with one shoulder thrown slightly forward and a little higher than the other. This was true in Mr. D.'s case; it is due to a slight lateral curvature of the spine. On this subject Mr. Treves says (International Encyclopedia of Surgery): "In certain cases there may be some slight lateral deviation of the spine in addition to the antero-posterior displaçement. This condition would appear to be met with only in the lumbar and dorso-lumbar regions."

Ask the little one to pick up a coin or handkerchief from the floor and observe the manner in which the stooping is accomplished ; instead of bending over with the ease and grace of a healthy child, there is an awkward squat, all the flexion necessary to enable the child to reach the floor being performed at the knees and hips, while the spine remains perfectly rigid.

In every motion which the child executes, there is the same careful avoidance of everything which will jar or bend the spine. The rigidity of the spine, which is such a characteristic symptom early in the disease, is one of nature's conservative processes. It is caused by the contraction of the muscles attached to the ribs and spine. This muscular contraction keeps the spine from bending at the seat of disease, acting as splints do on a broken limb. The fixation caused by this rigidity extends some distance above and below the diseased area. The rigidity of the spine, present very late in Pott's disease, and also in those cases in which a cure has been effected, is due to bony ankylosis. Greater diagnostic importance attaches to this early rigidity than to any other single symptom. "In examining a young child, it is most convenient to have it placed flat upon its face, and then, on lifting up the lower limbs and moving them (together with the pelvis) in various directions, with the unoccupied hand placed upon the back, any rigidity of the column can be soon estimated." Thus says Mr. Frederick Treves in a most excellent article on Pott's disease in the International Encyclopedia of Surgery. I quote this merely to condemn it, for I regard it as most pernicious advice. It is easy to conceive that in a child already strumous, the wrenching and twisting necessary to carry out Mr. Treves' method could readily cause an exacerbation of the existing inflammation, or produce a new focus of disease in another part of the spinal column. Besides, all that it is necessary to know about the rigidity can be ascertained by seeing the child stoop, by watching it rise from a recumbent position, and by observing the peculiar gait as it walks.

The general condition, appearance and behavior of the child will prove of great assistance in reaching a diagnosis.

Children who are the subjects of Pott's disease are very often well nourished in the sense of having a sufficient amount of fat upon the limbs and body, but on feeling their flesh it is found to be soft and flabby; this is generally so noticeable that the parents often call the doctor's attention to it. While this alone is in no way diagnostic of Pott's disease, occurring as it does in many other conditions, yet taken in conjunction with the other symptoms, it is of a certain value; it indicates a lowered vitality, such as is found in strumous subjects.

The general appearance and behavior of the child have already been sufficiently discussed. The facial expression of these little patients is often very characteristic; it is an expression of care, anxiety and watchfulness; and if there has been much suffering the facial lines are deepened, giving the child's face an old and quaint appearance.

If, in addition to the symptoms above enumerated, there is an abscess presenting in the neck, pelvis, groin or lumbar region, the diagnosis can be made all the more easily. There are certain diseases giving symptoms somewhat similar to those in the early stage of Pott's disease, and they must therefore be differentiated from it. These are rickets, lateral curvature of the spine, aortic aneurisms and hysteria. In rickets there is often an anteroposterior curvature of the spine, but it differs from the deformity of Pott's disease in this, it has no prominent "knuckle," and it can be straightened out by moderate extension. In addition, the child will present some of the other well-known symptoms of rickets, such as the large square head, the "beading" of the ribs, the enlargement of the radial epiphyses, delayed dentition, sweating about the head, etc.

Lateral curvature of the spine existing alone can be very easily differentiated from Pott's disease, but where a slight lateral curvature *complicates* Pott's disease, as in the case I have narrated, careful attention must be given to all the symptoms in order to reach an accurate diagnosis.

An aneurism of the aorta may so press on the bodies of the vertebræ as to erode them and cause an angular curvature of the spine. This condition must be diagnosticated by the age of the patient (aneurism usually occurs in middle life), the presence of atheromatous changes in the arteries, and by the physical signs which an aneurism gives on percussion and auscultation.

One of the protean manifestations of hysteria is the condition known as "hysterical spine." This can be diagnosticated by the much greater spinal tenderness than is ever present in Pott's disease, by the fact that this tenderness often extends along the whole length of the spinal column and by the co-existence of other hysterical symptoms.

TREATMENT.—On this subject I will only mention a few practical points. There is no question in my mind but that the plaster of Paris jacket, with or without the jury-mast (according to the location of the carious process), is the easiest, cheapest and best treatment in the vast majority of cases. To physicians who live in the country or in small towns it is really about the only available method, for it is very difficult for an instrument-maker to fit a spinal brace on a patient unless the measurements are taken by some one who is expert in the business.

In suspending adults preparatory to putting on the plaster jacket, great care should be taken not to draw them up too high. By producing too much extension of the spine we do an injury to the diseased spot, and also incur the risk of breaking up any solidification which may have begun. Adults not infrequently suffer considerable shock from the operation. I have known one patient, a young lady 21 years old, to faint during the suspension; another adult required three days to recover from the effects of it.

Children, as a rule, bear suspension very well, but with them also we must exercise great judgment as to the degree of extension.

Both with adults and children the operation should be performed with all the celerity possible. If there are excoriations of the skin of the back, or if there is a marked projection backward of the spinous processes endangering the safety of the skin, these parts should be protected from the direct pressure of the plaster by strips of piano-felt. The crests of the ilia should be covered by a layer of cotton wool, and the "dinner-pad" must not be forgotten under any circumstances. A seamless and sleeveless knit shirt is the best kind to have next the skin, for it does not allow the formation of folds or wrinkles.

